# Two cases of carina resection for bronchogenic tumor with lung parenchyma sparing: A brief report

**DOI:** 10.1016/j.ijscr.2019.12.007

**Published:** 2019-12-16

**Authors:** Beatrice Aramini, Gening Jiang, Jiang Fan

**Affiliations:** a University of Modena and Reggio Emilia, Modena, Italy; b Department of Thoracic Surgery, Shanghai Pulmonary Hospital, Tongji University, Shanghai, China

**Keywords:** CR, carina resection, CT, computed tomography, 18F FDG PET/CT, 18F-labeled fluoro-2-deoxyglucose Positron emission tomography–computed tomography, Lung sparing, Carina resection, Bronchogenic tumor, Lung carina tumor

## Abstract

**Introduction:**

Carinal resection (CR) with or without lung parenchyma sparing is one of the challenging issues in thoracic surgery and performed rarely. It is usually not used for the difficulty of the technique, or, in the majority of the situations, it is replaced by pneumonectomy when the patient conditions and the tumor characteristics allow the radical surgical approach. The difficulty includes the dissection of the trachea and main bronchi, the resection of the carina and the reconstruction of the trachea and bronchus. In spite of the knowledge of the technique, one of the most important problem is the incidence of postoperative complications which is higher compared to standard resections.

**Clinical cases:**

We show two cases of carina resection for a bronchogenic tumor with lung parenchyma sparing and good postoperative outcomes.

**Conclusion:**

Our results demonstrate that this type of surgery is possible in selected centers with good quality of life for the patient after surgery.

## Highlights

• Carinal resection with lung parenchyma sparing is a challenging issue in thoracic surgery.
• It is usually not used for the difficulty of the technique.
• It is replaced by pneumonectomy when the patient conditions allow this approach.
• Complications after carina resection are quite frequent.
• A high expertise and good coordination are mandatory to prevent severe complications.

## 1 Introduction

Cancer involving the carina without systemic or lymphatic metastases are uncommon but not rare [[1], [2], [3]]. Most of these patients were diagnosed at an advanced stage and were likely to be no longer candidates for surgical resection [1]. However, in not so low number of centres, even in local tumor of the carina, surgeon chooses for the option of pneumonectomy, if the patients’ conditions are favourable [4]. However, if surgery is not the best choice due to the limited pulmonary function or for the patient comorbidities, some thoracic surgeon sends directly the patient to the attention of the oncologist for medical treatment [5]. On the other hand, this choice is not so rarely adopted also in case of possibility to perform carina resection. In middle-low volume centres infact, this kind of surgery is not performed, for the low experience of the surgeons and the consequent high frequency of postoperative comorbidities. As for our experience, we show two cases of carina resection for a bronchogenic tumor with lung sparing. Our message is to show that carina resection in selected patients is possible with no complications after surgery and good results after 6 months of follow up. Our cases could highlight the importance to choose a high volume center for this kind of surgical resection, which could guarantee satisfying results and an acceptable quality of life for the patient after surgery. The work has been reported in line with SCARE criteria [6].

## 2 Case 1

**Fig. 1:**
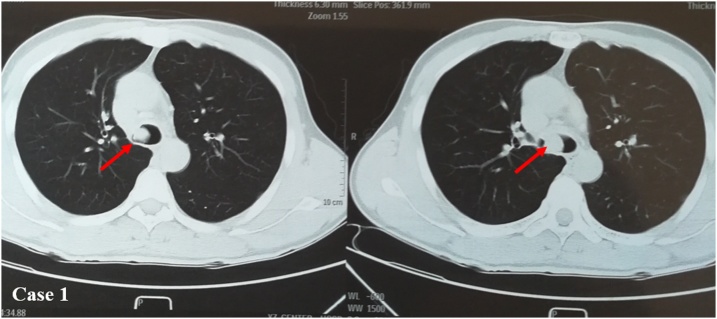
**Case 1. Chest CT shows** tumor infiltration between carina and the right main bronchus (see the arrow).

A 27 years old male patient suffering from cough and discomfort of throat for half a month was admitted to our Hospital. He referred a smoking history of 5–10 cigarettes per day for 5 years. Chest computed tomography (CT) showed a tumor infiltrating the entire wall of the carina at the level of the main right bronchus (Fig. 1). ^18^F-labeled fluoro-2-deoxyglucose Positron emission tomography–computed tomography (^18^F FDG PET-CT) showed a mild metabolic area at the level of the carina with no lymph nodes activity. A malignancy was suspected. No other metabolic activity was highlighted. Brain MRI showed no abnormalities. Preoperative assessments revealed a good pulmonary function test and no other comorbidities were declared by the patient. Tracheoscopy revealed a neoplasm in the opening of the right main bronchus, and pathology revealed highly differentiated mucoepidermoid carcinoma. He received a tracheal carina resection at the level of the right main bronchus and reconstruction with lung parenchyma preservation through a posterolateral thoracotomy. The operation time was 3 h and 40 min. Intraoperative bleeding was less than 200 ml. He was discharged 7 days after operation with no complications. Postoperative pathology revealed a 1.2 cm low-grade mucoepidermoid carcinoma involving the entire wall of the carina. The distal and proximal stump were found negative. The 2, 4, 7 and 10 lymph nodes stations were negative. After surgery patient has been sent to the oncologist for clinical monitoring. Bronchoscopy after 6 months from surgery showed a moderate stenosis at the level of the anastomosis. A biopsy was performed showing a granuloma treated by laser with completely effective result. No sign of recurrence of granuloma after 3 months from the laser procedure, as well as no sign of recurrence after 6 months from surgery.

## 3 Case 2

**Fig. 2:**
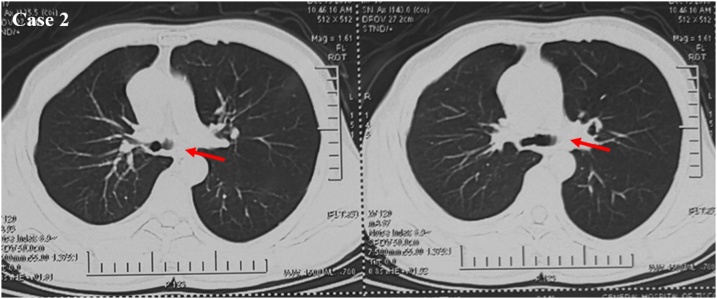
**Chest CT in case 2.** Tumor infiltrates the middle part of the carina closed to the left main bronchus (see the arrow).

A 43 years old male patient with cough and bloody sputum for a week was admitted to our Hospital. Chest CT showed a solid mass between the tracheal carina involving mainly the left main bronchus (Fig. 2). A malignant mass was suspected. ^18^F FDG PET-CT showed a high metabolic area at the level of the carina with no lymph nodes involvement. Tracheoscopy confirmed a neoplasm in the tracheal carina at the level of its bifurcation with the left main bronchus. Preoperative assessments revealed a good pulmonary function test. The patient undergone tracheal carina resection at the level of the left main bronchus and reconstruction with no lung resection through a posterolateral thoracotomy. No complications after surgery. Postoperative pathology revealed a 2 cm sarcomatous high-grade carcinoma. The distal and proximal stump were negative. The 3 and 10 lymph nodes stations were negative. After surgery patient has been sent to the oncologist for treatment and follow up. After 6 months from surgery no significant abnormalities or signs of recurrence were noted.

## 4 Discussion and conclusion

**Fig. 3:**
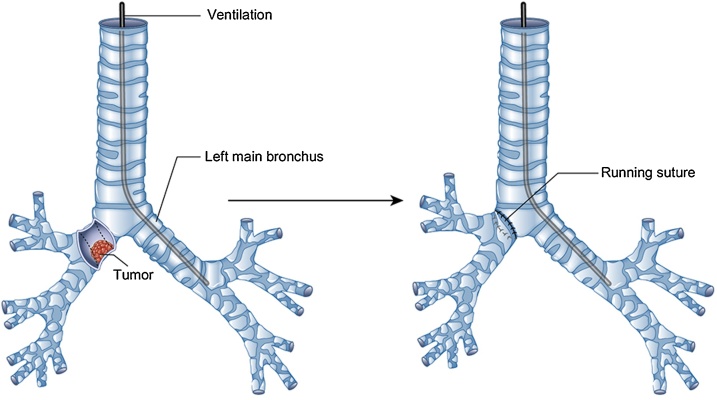
**Operative technique and ventilation in case 1.** The tracheal tube was pushed down into the left main bronchus and the surgeon proceeded with the resection of the tumor and subsequent anastomosis.

**Fig. 4:**
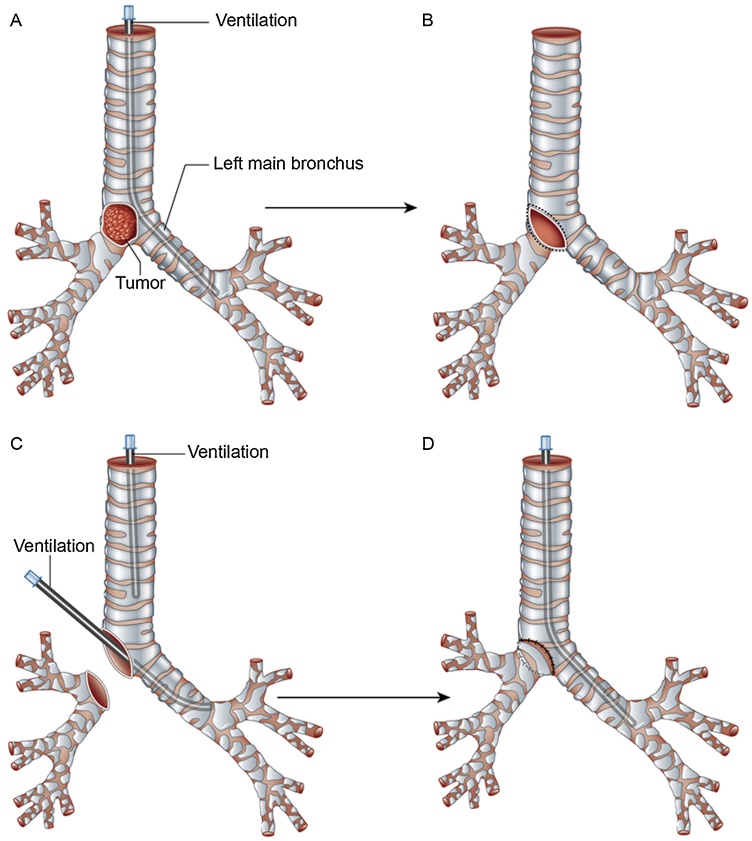
**Operative technique and ventilation in case 2. A–B.** The tumour infiltrated the middle part of the carina closely to the left main bronchus. The tumor was resected. **C–D.** The ventilation tube was then pushed through the right main bronchus into the left main bronchus to guarantee patient ventilation, as soon as the surgeon completes the tumor resection. During the anastomosis, the tube stayed in this position until the surgeon completes the bronchial anastomosis. After the anastomosis, a tracheal tube has been replaced.

The most common approach for carina resection is right thoracotomy through the fourth or fifth intercostal space. Left side approach due to left aortic arch is difficult or sometimes impossible [7]. However, as we showed in our brief report, the posterolateral thoracotomy may be a good choice to improve the view, especially for big tumor mass infiltrating the carina and the left main bronchus. Bilateral sub-mammary trans-sternal “clamshell” thoracotomy and median sternotomy are other ways to approach carina in selected patients [8]. Recently, video-assisted thoracoscopic tracheal resection and carinal reconstruction has also been reported, but this is mainly performed by expert thoracic surgeon and in high volume centres where this technique is quite frequently adopted [9]. With regards of the carina reconstruction techniques, various approaches have been proposed. They all depend in the extent of the resected trachea, left and right main bronchus [7]. Ventilation is another important step of this technique. In particular, in cases of tumor infiltrating one main bronchus, it is quite comfortable to resect the tumor closed to carina and make the anastomosis (Fig. 3), although in case of carina infiltration and in very selected cases, it is possible to resect the tumor with an accurate tracheal ventilation process set by the anaesthesiologist (Fig. 4). Infact, these steps needed to be manage by expert anaesthesiologists’ team coordinated by the surgeon.

**Fig. 5:**
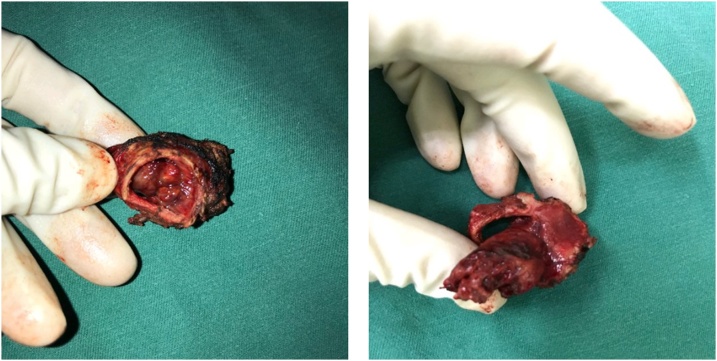
**Carina resection in case 2.** The tumor is in the middle of the carina.

On the other hand, carina resection (Fig. 5) is considered a good option to pneumonectomy or lobectomy, especially in cases where the comorbidities or pulmonary functional test not allowed to perform lung parenchyma resection. We believe that, if the preoperative radiological assessments show no lymph nodes infiltration or other suspicious tumor metastatic infiltrations, a carina resection with lung parenchyma sparing may be considered as the best choice, even in high grade tumor, as we showed for sarcomatoid mass described in case 2. The possibility to choose carina resection option instead of pneumonectomy is mainly based of surgeon experience, although a very accurate clinical and radiological assessment, before defining the surgical approach, is mandatory as guarantee for satisfying results, especially to prevent postoperative complications. In conclusion, our cases show the possibility to perform carina resection with lung sparing in low grade tumors as well as for high grade tumors, as we showed for case n.2. In both situations, the choice must be driven by expert surgeon in order to prevent complications as well as short term recurrence. Our report is the basement to set further studies regarding carina resection with or without lung parenchyma surgery. Further studies need to be plan in order to compare long term results between the cases of carina resection associated with pneumonectomy or lobectomy and the other cases with lung sparing, for low- and high-grade lung malignancies and no lymph nodes involvement. This will be very helpful for driving the surgical choices in case of resectable tumour infiltrating the carina, especially in patients with compromised clinical conditions.

## Funding

No funding.

## Ethical approval

For case series report NO ethical approval needs. Patient signed a consent for publishing the case report.

## Consent

Patients signed a consent for the publication of this case report.

## Author contribution

BA and JF wrote the case report. The other Authors read and revised the case report.

## Registration of research studies

Ethical Board approval is not required for case reports in our Center.

## Guarantor

Prof. Jiang Fan is the Guarantor of this case report.

## Provenance and peer review

Not commissioned, externally peer-reviewed

## Declaration of Competing Interest

The Authors have no financial and personal relationships to disclose.
